# Treatment modalities favoring outcome in well-differentiated neuroendocrine tumors G3

**DOI:** 10.3389/fendo.2023.1285529

**Published:** 2024-01-08

**Authors:** Martina Hinterleitner, Ruben Pfeiffer, Nils F. Trautwein, Bence Sipos, Stephan Singer, Silvio Nadalin, Alfred Königsrainer, Ulrich M. Lauer, Christian la Fougère, Lars Zender, Clemens Hinterleitner

**Affiliations:** ^1^ Department of Medical Oncology and Pneumology (Internal Medicine VIII), University Hospital Tuebingen, Tuebingen, Germany; ^2^ European Neuroendocrine Tumor Society (ENETS) Center of Excellence, University Hospital Tuebingen, Tuebingen, Germany; ^3^ German Research Foundation Deutsche Forschungsgemeinschaft (DFG) Cluster of Excellence 2180 ‘Image-Guided and Functional Instructed Tumor Therapy’ (iFIT), University of Tuebingen, Tuebingen, Germany; ^4^ Department of Nuclear Medicine and Clinical Molecular Imaging, University Hospital Tuebingen, Tuebingen, Germany; ^5^ Department of Pathology, University Hospital Tuebingen, Tuebingen, Germany; ^6^ Department of General and Transplant Surgery, University Hospital Tuebingen, Tuebingen, Germany; ^7^ German Cancer Consortium (DKTK), German Cancer Research Center (DKFZ), Tuebingen, Germany; ^8^ Cancer Biology and Genetics, Memorial Sloan Kettering Cancer Center, New York, NY, United States

**Keywords:** neuroendocrine tumor, grading, G3, somatostatin receptor, PET, treatment

## Abstract

**Introduction:**

Neuroendocrine neoplasms (NEN) are a rare and heterogenous group of tumors arising from neuroendocrine cells in multiple organs. Neuroendocrine tumors (NET) G3 encompass a small subgroup accounting for less than 10% of all neuroendocrine neoplasms. In contrast to NET G1 and G2 as well as neuroendocrine carcinomas (NEC), in NET G3 data on treatment and patient outcomes are still limited. Especially in a metastasized tumor stage, the role of surgery, peptide receptor radionucleotide therapy (PRRT), and systemic chemotherapy is not clearly defined.

**Methods:**

In this real-life cohort, we consecutively analyzed clinical outcome in NET G3 patients receiving different diagnostic and treatment.

**Results and discussion:**

We found that even metastasized NET G3 patients undergoing surgery, or receiving radiation, somatostatin analogues (SSA), and PRRT showed a clear survival benefit. Interestingly, all treatment regimen were superior to classical chemotherapeutic agents. In addition, somatostatin receptor (SSTR) PET-CT, FDG PET-CT, and repetitive biopsies were shown to be useful diagnostic and prognostic tools in NET G3. Our study demonstrates that patients with highly proliferative NET G3 might benefit from less aggressive treatment modalities commonly used in low proliferative NEN.

## Graphical Abstract

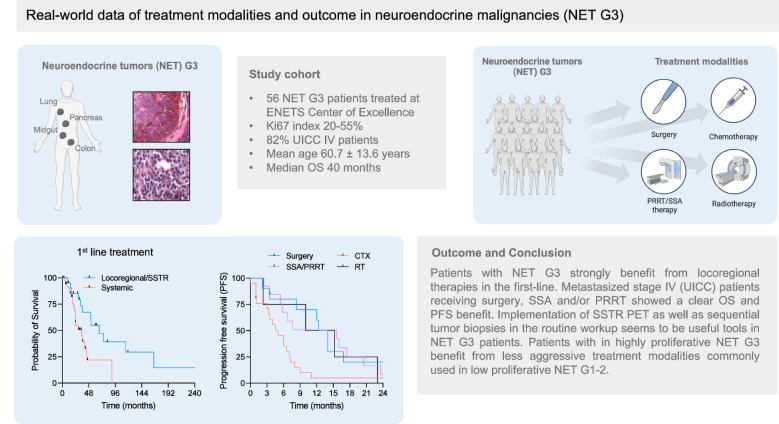

## Introduction

Neuroendocrine neoplasms (NEN) compromise a rare and heterogeneous group of tumors (1, 2). NEN arise from neuroendocrine cells of various organs, including gastrointestinal tract, pancreas, and lungs and are defined by the occurrence of specific histopathological markers such as synaptophysin, chromogranin A and CD56 (1–3). In addition to primary tumor site and tumor stage, the histopathological differentiation (G1–3) is one of the most relevant prognostic factors for overall survival (OS) in NEN (1, 2, 4, 5). The histopathological classification includes morphological criteria for well- and poorly differentiated tumors as well as a tumor grading based on Ki67 proliferation index and/or mitotic rate (3, 6). Tumor grading in gastroenteropancreatic (GEP) NEN is defined as low (G1, Ki67 <3%), moderate (G2, Ki67 3% to 20%), or high (G3 Ki67 >20%) (3, 6, 7). Among all GEP-NEN, the prevalence of G3 NET has been described from 5.6% to 8% (8–11). In the lung, four different neuroendocrine epithelial tumors arise: typical (0 or 1 mitosis per 2 mm^2^, and absence of necrosis), atypical carcinoid (2–10 mitoses per 2 mm^2^, and/or presence of necrosis), small cell neuroendocrine carcinoma (high mitotic counts), and large cell neuroendocrine carcinoma (high mitotic counts) (12).

However, especially neuroendocrine neoplasms G3 are extremely heterogenous, ranging from well-differentiated neuroendocrine tumors (NET) G3 to poorly differentiated neuroendocrine carcinomas (NEC) G3 (2). NET G3 are predominantly defined by a well-differentiated morphology and Ki67 index ranging from 20% mostly up to 55%, whereas NEC G3 present with a poorly differentiated morphology (large-cell or small-cell type) and Ki67 index in the majority of cases above 55% (2, 6, 11, 13).

Since the subgroup of NET G3 encompasses less than 10% of all GEP-NEN, data on treatment and outcome are very limited (2, 14). Regarding survival, OS has been shown to be significantly longer compared with NEC G3 but shorter than in NET G1 and G2 (2, 3, 15). Due to an enormous heterogeneity in studies evaluating NEN G3 so far, even in limited, non-metastasized tumor stages, the role of surgery has not been defined and guidelines for different treatment modalities are largely missing (3, 16). Nevertheless, recent data show that surgical management of GEP-NEN G3 may lead to survival benefit in selected cases (16, 17). In advanced NET G3, the optimal first-line therapy is not defined, considering the paucity of prospective trials (2, 3). Platinum-based first-line therapies are commonly used; however, response rates in NEN with a Ki67 index below 55% are lower than those of NEN with a Ki67 index above 55% (3, 18). Chemotherapeutic regimens commonly used in NET G2, especially capecitabine combined with temozolomide (CAPTEM), seem to be an additional therapeutic option for first-line treatment of NET G3 (2, 3, 19, 20). According to the high expression of somatostatin receptor (SSTR) commonly observed in NET G3, also treatment with somatostatin analogues (SSA) and/or peptide receptor radionucleotide therapy (PRRT) has been described, although SSTR PET imaging upfront and short-term interval imaging to assess disease control are needed (3, 19, 21–25). Further therapeutic options include mTOR inhibitors like everolimus and tyrosine kinase inhibitors such as sunitinib (3, 26–28). Of note, clinical trials investigating the benefit of immunotherapy in NET G3 were disappointing so far (3, 29, 30).

Given the lack of consecutive clinical data in NET G3 so far, we here evaluated a real-live cohort of NET G3 patients regarding clinical characteristics, outcome, and different treatment regimen. Interestingly, we found that even metastasized NET G3 patients undergoing surgery or receiving SSA/PRRT showed a significant survival benefit compared with patients treated systemically with classical chemotherapeutic agents. In addition, SSTR PET-CT, FDG PET-CT, and repetitive biopsies were shown to be a useful prognostic tool in neuroendocrine malignancy NET G3. Our data provide first evidence that even patients with highly proliferative NET G3 benefit from less aggressive treatment modalities commonly used in low proliferative NET G1–2.

## Materials and methods

### Study population

Patients with the histopathological confirmed diagnosis of a neuroendocrine tumor G3 at the time point of primary diagnosis, treated at the ENETS Center of University Hospital Tuebingen, were included in this retrospective observational study. Between January 2016 and October 2022, 61 patients (39 male, 22 female, mean age 57.89 ± 13.14 years) with NET G3 at the time point of primary diagnosis received diagnostics and treatment at University Hospital Tuebingen and were consecutively included in this study. Five out of these 61 patients had mixed neuroendocrine–non-neuroendocrine (MiNEN) neoplasms with a predominant neuroendocrine part and Ki67 index above 20%. Since the outcome of this patients might be driven by the adenocarcinoma component, we excluded all MiNEN patients from further analysis. All histopathological diagnoses were at least confirmed by one reference pathologist (B.S. and/or S.S.). Treatment decisions were based exclusively on a consent made in the interdisciplinary tumor board of the University Hospital Tuebingen. Patient characteristics in detail are shown in **Table 1**. Treatment schemes in detail are presented in **Table 2** and **Supplementary Table 1**. The study was approved by the IRB (ethics committee of the Faculty of Medicine of the Eberhard Karls University Tuebingen and of the University Hospital Tuebingen) (reference number 362/2021BO2) and was conducted in accordance with the Declaration of Helsinki.

**Table 1:** Patient characteristics of the NET G3 cohort.

Patient characteristics	Total (n=56)
Sex	
male, n (%)	35 (62.5)
female, n (%)	21 (37.5)
**Age in years at first diagnosis**, mean – yr. ± SD (range)	60.66 ± 13.58
**OS**, mean	40
TNM classification, n (%)	
Tumor	
Tx	3 (5.36)
T0	12 (21.43)
T1	2 (3.57)
T2	11 (19.64)
T3	15 (27.87)
T4	12 (26.79)
Node	
Nx	10 (17.86)
N0	12 (21.43)
N+	34 (60.71)
Metastases	
M0	10 (17.86)
M1	46 (92)
UICC classification, n (%)	
I	2 (3.57)
II	2 (3.57)
III	6 (10.71)
IV	46 (92)
**Ki67 index at first diagnosis**, mean – % ± SD (range)	33.41 ± 8.67
Primary tumor site, n (%)	
pancreas	19 (33.93)
CUP	12 (21.43)
lung	6 (10.71)
appendix	3 (5.36)
midgut	5 (8.93)
colorectal	4 (7.14)
miscellaneous	7 (12.5)

CUP, Cancer of unknown primary; G3, grading 3; m, months; M, metastases; n, number; N, node; NEN, neuroendocrine neoplasm; NET, neuroendocrine tumor; OS, overall survival; SD, standard deviation; T, tumor; UICC, Union for International Cancer Control; yr., years; %, percentage.

**Table 2:** Treatment schemes of the NET G3 cohort for first-line, second-line and third-line therapy.

Treatment schemes	Patients
1st line therapy regimen, n (%)	
Surgery of the primary tumor site	13 (23.21)
Surgery of metastases	4 (7.14)
SSA	5 (8.93)
PRRT	7 (12.5), including 3 in combination with radiosensitizing (CAPTEM)
SIRT	1 (1.79)
RT of metastases	3 (5.36)
Systemic therapies (other than SSTR-directed)	22 (39.29)
Platinum/etoposide	8 (14.29)
CAPTEM	4 (7.14)
FOLFOX	3 (5.36)
FOLFIRINOX	2 (3.57)
Streptozotocin/5-FU	1 (1.79)
Carboplatin/paclitaxel	1 (1.79)
Everolimus	1 (1.79)
No therapy	2 (3.57)
2nd line therapy regimen, n (%)	
Surgery of the primary tumor site	5 (8.93)
Surgery of metastases	5 (8.93)
SSA	8 (14.29)
PRRT	8 (14.29), including 3 in combination with radiosensitizing (CAPTEM)
SIRT	1 (1.79)
RT of the primary tumor site	2 (3.57)
RT of metastases	4 (7.14)
Systemic therapies (other than SSTR-directed)	19 (33.93)
Platinum/etoposide	10 (17.86)
CAPTEM	3 (5.36)
CAPTEM/bevacizumab	1 (1.79)
FOLFIRI	1 (1.79)
Topotecan	1 (1.79)
Gemcitabine	1 (1.79)
Carboplatin/paclitaxel/etoposide	1 (1.79)
3rd line therapy regimen, n (%)	
Surgery of the primary tumor site	4 (7.14)
Surgery of metastases	5 (8.93)
SSA	12 (21.43)
PRRT	4 (7.14), including 2 in combination with radiosensitizing (CAPTEM)
RT of metastases	2 (3.57)
Systemic therapies (other than SSTR-directed)	18 (32.14)
Platinum/etoposide	2 (3.57)
CAPTEM	6 (10.71)
FOLFOX	1 (1.79)
FOLFOX/bevacizumab	3 (5.36)
Topotecan	1 (1.79)
Nivolumab/ipilimumab	1 (1.79)

1^st^, first; 2^nd^, second; 3^rd^, third; 5-FU, 5-fluorouracil; CAPTEM, capecitabine/temozolomide; FOLFIRI, irinotecan; 5-fluorouracil, leucovorin; FOLFIRINOX, oxaliplatin, irinotecan, 5-fluorouracil, leucovorin; FOLFOX, oxaliplatin, 5-fluorouracil, leucovorin; G3, grading 3; n, number; NET, neuroendocrine tumor; PRRT, peptide receptor radionucleotide therapy; RT, radiotherapy; SIRT, selective internal radiotherapy; SSA, somatostatin analogue; SSTR, somatostatin receptor; %, percentage.

### Collection of data

For each patient included in the study, the following parameters were evaluated: sex, age, primary diagnosis, primary tumor site, overall survival (OS) after initial histological diagnosis of NET G3, progression-free survival (PFS) after first-line treatment, TNM classification, UICC stage, histopathology including Ki67 index at first diagnosis and in the course of disease, treatment schemes in the first line, second line, and third line, and further therapy lines including locoregional treatment (surgery of primary tumor site and/or metastases, radiotherapy of primary tumor site and/or metastases, SSTR targeting therapies (somatostatin analogues (SSA) and/or peptide receptor radionucleotide therapy (PRRT)), and further systemic treatments (cytotoxic chemotherapy (CTX), mTOR inhibitor everolimus, tyrosine kinase inhibitors such as sunitinib, immunotherapy, and others). Additionally, laboratory parameters including lactate dehydrogenase (LDH) in U/L, neuron-specific enolase (NSE) in µg/L, chromogranin A (CgA) in µg/L, serotonin in µg/L, absolute neutrophile count (ANC), absolute lymphocyte count (ALC), hemoglobin (Hb) level in g/dL, and platelet (PLT) count were determined at timepoint of first diagnosis and frequently in the course of disease, especially in the case of treatment change. Furthermore, performance of somatostatin receptor (SSTR) PET imaging and/or fluorodeoxyglucose (FDG) PET, body mass index (BMI) in kg/m^2^, and the occurrence of diabetes were evaluated.

### Statistical analysis

Descriptive statistics were applied to characterize patients according to sex, age, primary diagnosis, primary tumor site, TNM classification, UICC stage, histopathology including Ki67 index at first diagnosis and in the course of disease, and treatment schemes in the first line, second line, third line, and further therapy lines. Prior to performing any statistical test, we tested for normal distribution using the D’Agostino & Pearson test. For continuous variables, Student’s t test, Mann–Whitney U test, one‐way ANOVA, and Friedman’s test were used, and chi‐squared test or Fisher’s exact test was used for categorical variables. If significant differences by one-way ANOVA were found, groupwise comparison was done (Tukey’s multiple comparison test). If significant differences by Friedman’s test were found, Dunn’s multiple comparisons test was used. OS and PFS, including the median, were calculated using the Kaplan–Meier method. Hazard ratios (HRs) were determined using Cox regression analysis. OS was calculated from the date of primary diagnosis. All statistical tests were considered statistically significant when p was below 0.05. Statistical analysis was performed using GraphPad Prism (v.9.1.2).

## Results

### Treatment modalities favoring outcome in neuroendocrine tumors G3

**Figure 1:**
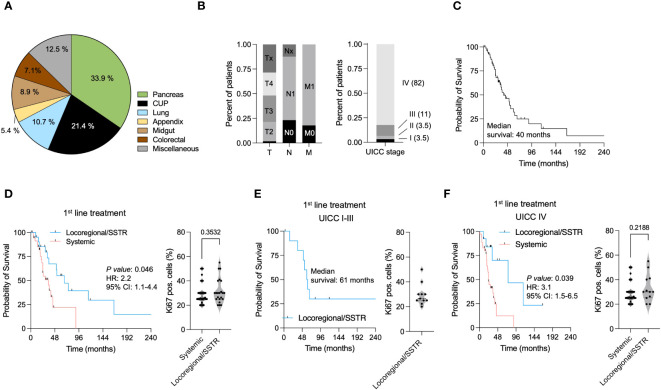
Treatment modalities favoring outcomes in neuroendocrine malignancies (NET G3). **(A)** Distribution of different histopathological subtypes in the NET G3 study cohort. **(B)** Tumor staging (T,N,M) and UICC stages at primary diagnosis. **(C)** Kaplan–Meier curves estimates for OS (in months) in the entire study cohort of NET G3 patients (n=63). **(D)** Kaplan–Meier curves estimates of OS (in months) in patients receiving a locoregional/SSTR directed (blue) and systemic treatment (red) as first line therapy. Ki67 pos. cells in patients receiving a locoregional/SSTR directed and systemic treatment. **(E)** Kaplan–Meier curves estimates of OS (in months) in UICC I-III patients receiving a locoregional treatment. Ki67 pos. cells in patients receiving a locoregional/SSTR targeting treatment. **(F)** Kaplan–Meier curves estimates of OS (in months) in UICC IV patients receiving a locoregional/SSTR targeting (blue) and systemic treatment (red) as first line therapy. Ki67 pos. cells in patients receiving a locoregional/SSTR directed and systemic treatment.

Even though treatment algorithms are well established in low-grade neuroendocrine tumors (NET G1 and G2, Ki67 pos. cells <1%–20%) and highly aggressive NEC, general guidelines for diagnostic workup and subsequent therapy are largely missing in the group neuroendocrine tumors G3 (Ki67 pos. cells > 20%). In order to better define a reasonable diagnostic workup and optimal treatment for this rare subgroup of NET G3 patients, we subsequently analyzed 61 patients with histopathologically confirmed diagnosis of NET G3 treated at the ENETS Center of Excellence Tuebingen in between January 2016 and October 2022. 36.1% pancreatic neuroendocrine tumors (pNETs) were the most common subgroup in our patient cohort, followed by carcinoma of unknown primary (CUP) NET (19.7%), neuroendocrine tumors of the lung (9.8%), and neuroendocrine tumors with gastrointestinal origin (**Figure 1A**). The majority of patients showed advanced disease stages with lymph node involvement or metastasis at primary diagnosis. Subsequently, 82% of patients were classified as UICC stage IV (**Figure 1B**). Comparable with other studies in NET G3, the median survival in the entire cohort was 40 months (**Figure 1C**).

To further investigate the influence of different treatment modalities on OS in NET G3, we analyzed patients receiving a locoregional or SSTR directed and systemic first-line treatment. Locoregional or SSTR targeting treatment included surgery, radiotherapy, SSA therapy, and PRRT. Systemic treatment comprised different chemotherapeutic regimen as cis-/carboplatin and etoposide, capecitabine/temozolomide (CAPTEM), or 5-FU-based treatments (**Table 2**). Of note, patients receiving a locoregional or SSTR targeting treatment showed a significantly prolonged OS compared with patients with a systemic treatment (HR: 2.9, 95% CI: 1.4–6, *p* = 0.014, **Figure 1D**). Since higher proliferation rates in this heterogenous cohort of NET G3 might impact the previous observation, we analyzed Ki67 pos. cells in both groups (patients treated with locoregional or SSTR targeting treatment and patients receiving other systemic treatments). However, we did not observe significant differences within the tumor proliferation rates in both groups (**Figure 1D**). Taking into account that locoregional treatment in UICC I–III stages has curative potential, we performed subgroup analyses in NET G3 patients presenting with UICC I–III vs. UICC IV stages. All patients with UICC I–III received locoregional treatment in the first line, and compared with the entire cohort, we observed a prolonged median survival of 61 vs. 40 months (**Figure 1E**). Similar to the entire cohort, in patients with UICC IV, we observed a significant OS benefit for patients receiving a locoregional treatment (HR: 2.4, 95% CI: 1.1–5.2, *p* = 0.035, **Figure 1F**). Again we did not observe a difference in tumor proliferation rates within both groups (**Figure 1F**).

**Figure 2:**
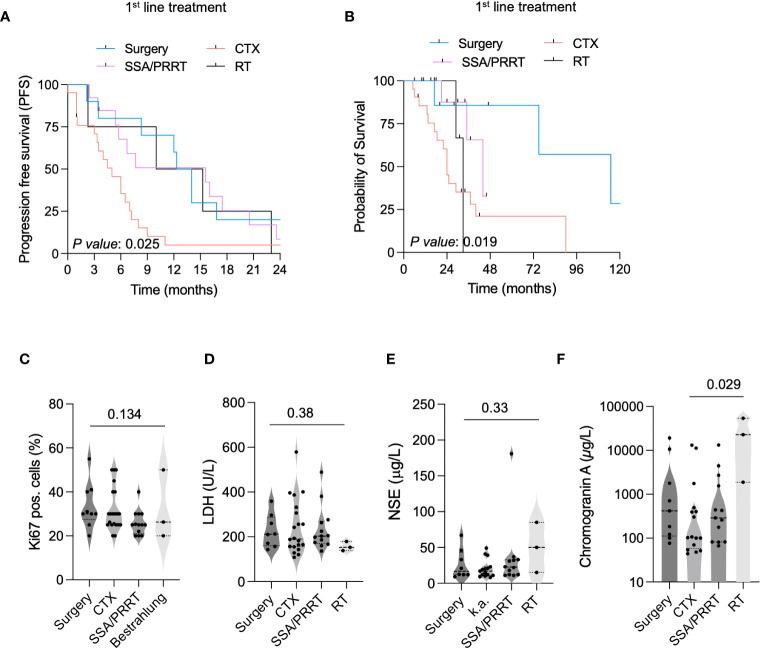
Locoregional treatments increase survival in neuroendocrine malignancies (NET G3). **(A)** Kaplan–Meier curves estimates of PFS (in months) in patients receiving surgery, CTX, SSA/PRRT or RT as first-line therapy. **(B)** Kaplan–Meier curves estimates of OS (in months) in NET G3 patients receiving surgery, CTX, SSA/PRRT or RT as first-line therapy. **(C)** Ki67 pos. cells in different treatment groups. **(D)** LDH levels (U/L) in different treatment groups. **(E)** NSE levels (µg/L) in different NET G3 treatment groups. **(F)** Chromogranin A level (µg/L) in different treatment groups.

To further dissect the influence of the different first-line treatment modalities on PFS and OS in NET G3, we performed subgroup analyses in patients receiving surgery, radiotherapy, SSA and/or PRRT, or other systemic treatment. With a median PFS of 15.6 months for patients treated with SSA and/or PRRT and 13.2 months for patients who underwent surgery, both groups showed prolonged PFS compared with patients treated with radiotherapy (10 months) or chemotherapy (5 months) (**Figure 2A**). Overall survival analysis revealed a benefit for patients undergoing surgery (median OS: 115 months), followed by patients receiving SSA/PRRT (median OS: 44 months) and patients with radiotherapy (median OS: 33 months). Patients with a systemic treatment showed only a median OS of 24 months (**Figure 2B**). In order to identify cofactors influencing our observations, we analyzed tumor proliferation rates (**Figure 2C**), LDH level (**Figure 2D**), NSE level (**Figure 2E**), and CgA concentrations (**Figure 2F**) at primary diagnosis. We did not observe differences in between Ki67 pos. cells, LDH level, or NSE level in the different groups. Interestingly, patients undergoing a radiotherapy showed an elevated CgA level (**Figure 2F**). In the second- or third-line treatment, SSA and/or PRRT remained to be associated with a prolonged OS (median OS: 40 months). Here, with a median OS of 24 months, surgery showed no benefit compared with systemic therapy (median OS: 24 months, **Supplementary Figure 1A**). In conclusion, NET G3 patients treated with locoregional treatment regimen including surgery and/or radiotherapy or SSTR targeting treatment (SSA and/or PRRT) in the first line showed a significant OS and PFS benefit. Interestingly, patients treated with aggressive systemic treatment regimen commonly used in highly aggressive NEC did not show OS or PFS benefit (**Supplementary Figure 1B**). Of note, all subgroups were balanced with regard to tumor proliferation rate (Ki67 level), LDH level, and NSE level. Since more prospective clinical data and a better accessibility of specific treatments including PRRT changed the treatment landscape of neuroendocrine tumors over time, we analyzed the use of different treatment strategies between 2016 and 2022. Interestingly in our small patient cohort, we were not able to identify a significant change in between the different treatment strategies over time (**Supplementary Figure 2A**). In addition, we did not obverse a significant association of the use of CTX, RT, SSR/PRRT, or surgery and patient age (**Supplementary Figure 2B**). Of note, larger patient cohorts and longer observation periods might be needed to adequately address this topic.

### SSTR and FDG PET-CT serve as prognostic factors in NET G3

**Figure 3:**
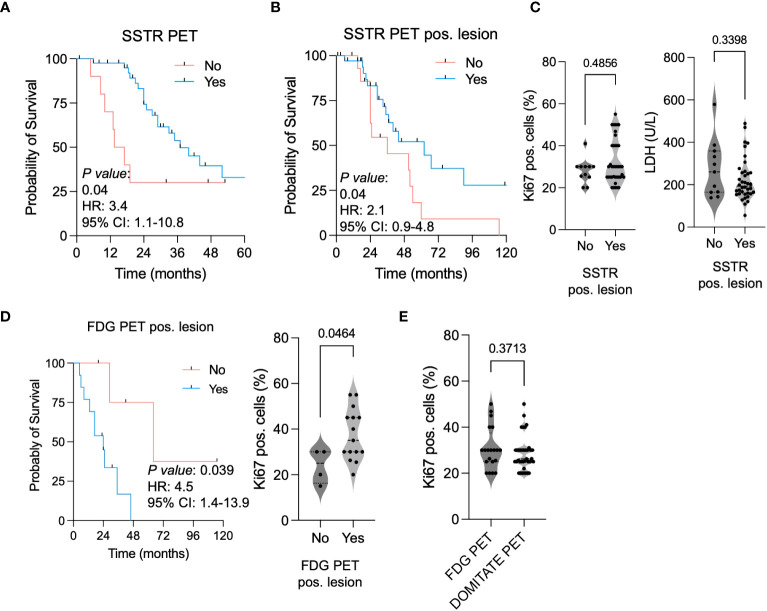
SSTR PET-CT favoring outcomes in neuroendocrine malignancies (NET G3). **(A)** Kaplan–Meier curves estimates of OS (in months) in patients receiving a SSTR PET-CT scan. **(B)** Kaplan–Meier curves estimates of OS (in months) in patients showing a SSTR PET-positive lesion. **(C)** Ki67 pos. cells and LDH level (U/L) in patients showing a SSTR PET-positive lesion. **(D)** Kaplan–Meier curves estimates of OS (in months) in patients showing a FDG PET-positive lesion. Ki67 pos. cells in patients with a FDG PET-positive vs. negative lesion. **(E)** Ki67 pos. cells in patients receiving a FDG PET-CT vs. SSTR PET-CT scan.

In a second step, we comparatively analyzed the diagnostic workup in our patient cohort. Even though SSTR PET-CT scans are not routinely performed in NET G3 patients, 79.2% of patients with UICC IV received a SSTR PET-CT scan at primary diagnosis. Taking into account that an SSTR-positive lesion is a prerequisite for a targeted therapy (SSA/PRRT), patients who received an SSTR PET-CT showed a significant better OS (HR: 3.4, 95% CI: 1.1–10.8, **Figure 3A**). In accordance with this observation, patients presenting at least one pos. lesion also showed a significant increased OS (HR:2.1, 95% CI: 0.9–4.8, **Figure 3B**). Of note, SSTR pos. lesions where not associated with decreased Ki67 or LDH level (**Figure 3C**).

In 39.6% of patients (UICC IV), an FDG-PET CT was performed at primary diagnosis. Patients with FDG-PET-positive lesions showed not only a higher Ki67 level (**Figure 3D**). A positive lesion was additionally associated with a shorter OS (HR: 5.4, 95% CI: 1.9–15.5) (**Figure 3D**). We did not observe a difference of Ki67 level in between patients receiving an SSTR- or FDG-PET-CT scan at primary diagnosis (**Figure 3E**). In conclusion, we observed that patients receiving an SSTR PET-CT scan at primary diagnosis and particularly patients with SSTR pos. lesions showed a clear OS survival benefit. FDG-CT scans efficiently identified patients with higher proliferative NET G3, and correspondingly FDG-PET pos. lesions were associated with a worse OS.

### Consecutive biopsies serve as a useful prognostic tool in NET G3

**Figure 4:**
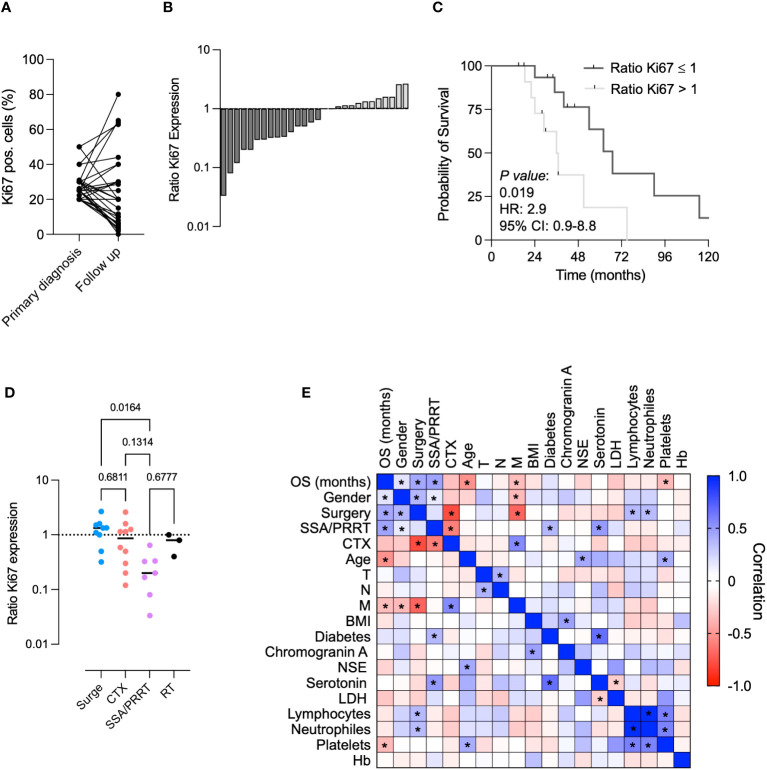
Consecutive biopsies serve as useful prognostic tool in NET G3. **(A)** Comparison of Ki67 pos. cells in primary vs. follow up biopsy. **(B)** Ki67 ratio (Ki67 pos. cells at primary diagnosis/Ki67 pos. cells in follow up biopsy) in all NET G3 patients with one consecutive biopsy. **(C)** Kaplan–Meier curves estimates of OS (in months) in patients showing a Ki67 ratio >1 vs. <1. **(D)** Ki67 ratios in different treatment conditions. **(E)** Multivariate comparative analysis for factors associated with a prolonged survival. * = p<0.05.

In 29 patients (46%), a subsequent biopsy of the primary tumor was performed. Overall, we observed a significant decrease in Ki67 pos. cells in the follow-up tumor biopsy (mean Ki67 pos. cells (%) initial biopsy 28.5 vs. 24.6 follow-up, **Figure 4A**). Even though the majority of patients showed a decrease in Ki67 levels, we identified a subgroup of patients with increased levels of Ki67. In order to further characterize these patients, we calculated the ratio of Ki67 pos. cells at primary diagnosis and Ki67 pos. cells in the follow-up biopsy. Here, we observed an overall decrease (ratio Ki67 expression <1) in 16 patients (55.1%), two patients (6.9%) showed no changes (ratio Ki67 expression = 1) and 11 (37.9%) showed an increase of Ki67 pos. cells over time (ratio Ki67 expression > 1, **Figure 4B**). Of note, Ki67 ratios were not dependent on the initial level of Ki67 pos. cells (**Supplementary Figure 3**). This suggests that treatment-associated changes in tumor proliferation rates were independent from the initial Ki67 level.

Interestingly, the ratio of the Ki67 expression (<1 vs. >1) served as a reliable prognostic marker to predict OS in our cohort of NET G3 patients (**Figure 4C**). We further used the Ki67 ratio to detect changes in tumor proliferation in different treatment modalities. Whereas patients who underwent a surgical procedure showed predominantly positive Ki67 ratios, we detected a significant decrease (Ki67 ratio <1) in patients treated with SSA and/or PRRT (**Figure 4D**). In patients treated with chemotherapy, we observed two populations (Ki67 ratio >1 and <1). This might reflect the overall response to the respective treatment. Patients receiving a radiotherapy showed a slight decrease of Ki67 ratios. Even though our patient samples were limited, we showed here that repetitive tumor biopsies and subsequent calculation of the Ki67 ratio can serve as a useful prognostic tool in NET G3.

Finally, we performed a multivariate analysis in our NET G3 cohort. Whereas gender (mean OS female: 71.6 months vs. mean OS male: 36.4 months), surgery, and SSA and/or PRRT were shown to be significantly associated with a prolonged survival, chemotherapy, age, the occurrence of metastasis, and elevated platelet counts were negative predictors for OS (**Figure 4E**).

In conclusion, our study implicates, that patients with NET G3 strongly benefit from locoregional therapies in the first-line. Interestingly, even UICC IV patients with surgery showed a clear OS and PFS benefit. In addition, SSA and/or PRRT had remarkable effects on OS and PFS. Taking this into account, the implementation of a SSTR PET-CT in the routine diagnostic workup seems useful in NET G3 patients. Finally, sequential tumor biopsies and concomitant Ki67 ratios might be useful as novel prognostic marker in NET G3.

## Discussion

Real-world data constantly analyzing patient survival and treatment outcome are curricle for the development of treatment guidelines in cancer. However, in rare and heterogenous tumor types large clinical data sets are missing. Neuroendocrine neoplasms encompass a rare and heterogeneous group of tumors arising from neuroendocrine cells in various organs (1, 4, 5). Within the group of NEN most data are existing for GEP-NET with low to moderate proliferation rates (NET G1-2) and neuroendocrine carcinomas (NEC) (1, 4, 31). In localized NEN surgical tumor resection is the best curative option (5, 32). For low NET G1-2 tumors expressing SSTR, SSA and/or PRRT show favorable response rates, significantly prolonging patient survival (5). For highly proliferative NEC platinum-based first-line therapies are well established and commonly used (5). However, due to limited availability of clinical data for the sparse subgroup of NET G3 (Ki67 >20%) no general treatment guidelines have been established so far. In our real-world cohort of 61 NET G3 patients we consecutively analyzed clinical characteristics, treatment schedules and patient outcomes.

Our patient cohort encompasses 59.1% NET with a gastroenteropancreatic origin, followed by CUP-NET and pulmonary NEN. This is in line with previous larger cohorts of NEN (1, 2, 20), confirming the representative nature of our study cohort. In our cohort only 18% of patients presented with a limited disease stage. All of these patients underwent surgery in the first line, leading to a 48 months recurrence-free survival of 81.8%. Even if these data are promising and in line with previous published data (32, 33), the majority of our patients (82%) presented with metastasis at primary diagnosis. Especially in this cohort various chemotherapeutic agents have been tested, with mixed results (20). In our cohort we observed that patients undergoing locoregional treatment or SSTR based therapies showed a significant survival benefit compared with patients receiving conservative chemotherapeutic therapy regimes including platinum/etoposide, CAPTEM, STZ/5-FU or FOLFOX. Of note, the Ki67 levels in both groups of patients didn’t differ significantly. This observation is remarkable, since in clinical practice NET G3 tumors showing higher proliferation rates are regularly treated according to the guidelines of high proliferative NEC (2, 3). However, the observation that surgery in patients presenting with metastasis can significantly prolong survival has made in several solid tumors including colorectal cancer, sarcomas and ovarian cancer (34–38). Our study shows that surgery in metastasized NET G3 patients represents a suitable treatment option, which is in line with previous results summarized by Holmager and colleagues (17). In addition, our data showed that SSA and/or PRRT, two treatment options commonly used for NET G1-2, showed a clear PFS and OS benefit in NET G3. This observation is in line with previous small cohorts evaluating SSA/PRRT in NET G3 (39). Due to the expected prolonged time to response compared with classical chemotherapeutic agents SSA and/or PRRT are still barley used in high proliferative NEN so far (3). The survival benefit of SSA and/or PRRT in our cohort is also reflected by the predictive value of the availability of a SSTR PET and the occurrence of SSTR pos. lesions.

In contrast, FDG PET positivity negatively correlates with OS, in accordance with previously published studies (40). Finally, our data reveal that consecutive biopsies and the determination of Ki67 and the ratio of Ki67 level (primary diagnosis/follow up) in NET G3 can serve as useful prognostic and potential predictive biomarker in NET G3. This is especially important in the subgroup of NEN, since an accelerated tumor proliferation clearly has therapeutic consequences (40). Overall we observed a decreased proliferation after treatment with conventional chemotherapy and SSA/PRRT. This is remarkable, since to our knowledge, SSA/PRRT in NET G3 has not yet been shown to decrease Ki67 levels significantly.

In conclusion, our study shows that NET G3 patients clearly benefit from treatment options commonly used for low proliferative NET G1-2. This might indicate that, even if NET G3 patients show higher proliferation rates (Ki67 level), the tumor biology is more comparable with NET G1-2 then to NEC (41, 42). Even if no consecutive clinical data comparing treatment regimen in this subgroups are existing so far, comparative genetic data reveal that NEC and NET G3 show two distinct genetic subgroups (42). Nonetheless, our study has some limitations. The retrospective characteristic of the study only allows for correlations rather than causal relationships between the individual factors. The small sample size might act as additional confounding factor here. Overall, larger prospective and multicenter studies are urgently needed to further investigate the role of surgical interventions, SSA/PRRT and radiation in patients with highly proliferative NET G3.

## Data availability statement

The raw data supporting the conclusions of this article will be made available by the authors, upon reasonable request.

## Ethics statement

The studies involving humans were approved by the Ethics Committee of the Faculty of Medicine of the Eberhard Karls University Tuebingen (reference number 362/2021BO2). The studies were conducted in accordance with the Declaration of Helsinki and the local legislation and institutional requirements. The participants provided their written informed consent to participate in this study.

## Author contributions

MH: Conceptualization, Data curation, Formal analysis, Investigation, Methodology, Project administration, Resources, Supervision, Validation, Visualization, Writing – original draft, Writing – review & editing. RP: Data curation, Formal analysis, Writing – original draft. NT: Data curation, Formal analysis, Investigation, Methodology, Writing – review & editing. BS: Investigation, Supervision, Writing – review & editing. SS: Data curation, Formal analysis, Investigation, Validation, Writing – review & editing. SN: Formal analysis, Methodology, Writing – review & editing. AK: Supervision, Validation, Writing – review & editing. UL: Funding acquisition, Methodology, Project administration, Supervision, Writing – review & editing. CF: Investigation, Supervision, Writing – review & editing. LZ: Conceptualization, Funding acquisition, Supervision, Writing – review & editing. CH: Conceptualization, Data curation, Formal analysis, Investigation, Methodology, Project administration, Resources, Supervision, Visualization, Writing – review & editing.

## Supplementary material

Supplementary Figure 1 Treatment outcomes in NET G3. **(A)** Kaplan–Meier curves estimates of OS (in months) in patients receiving surgery, SSA/PRRT or CTX in ≥ second line treatment. **(B)** Kaplan–Meier curves estimates of OS (in months) in NET G3 patients receiving CTX.

Supplementary Figure 2 Distribution of treatment modalities over time and patient age. **(A)** Distribution of treatment modalities over time. **(B)** Correlation of treatment modalities and patient age.

Supplementary Figure 3 Changes in Ki67 levels in NET G3. **(A)** Correlation of Ki67 levels at primary diagnosis and Ki67 ratios.

## References

[1] Yao JC Hassan M Phan A Dagohoy C Leary C Mares JE. One hundred years after "carcinoid": epidemiology of and prognostic factors for neuroendocrine tumors in 35,825 cases in the United States. J Clin Oncol (2008) 26(18):3063–72. doi: 10.1200/JCO.2007.15.4377 18565894

[2] Heetfeld M Chougnet CN Olsen IH Rinke A Borbath I Crespo G. Characteristics and treatment of patients with G3 gastroenteropancreatic neuroendocrine neoplasms. Endocr Relat Cancer (2015) 22(4):657–64. doi: 10.1530/ERC-15-0119 26113608

[3] Alheraki SZ Almquist DR Starr JS Halfdanarson TR Sonbol MB. Treatment landscape of advanced high-grade neuroendocrine neoplasms. Clin Adv Hematol Oncol (2023) 21(1):16–26. 36638352

[4] Rindi G Klöppel G Alhman H Caplin M Couvelard A de Herder WW. TNM staging of foregut (neuro)endocrine tumors: a consensus proposal including a grading system. Virchows Arch (2006) 449(4):395–401. doi: 10.1007/s00428-006-0250-1 16967267 PMC1888719

[5] Lepage C Bouvier AM Phelip JM Hatem C Vernet C Faivre J. Incidence and management of Malignant digestive endocrine tumours in a well defined French population. Gut (2004) 53(4):549–53. doi: 10.1136/gut.2003.026401 PMC1774002 15016750

[6] Nagtegaal ID Odze RD Klimstra D Paradis V Rugge M Schirmacher P. The 2019 WHO classification of tumours of the digestive system. Histopathol (2020) 76(2):182–8. doi: 10.1111/his.13975 PMC7003895 31433515

[7] Milione M Maisonneuve P Spada F Pellegrinelli A Spaggiari P Albarello L. The clinicopathologic heterogeneity of grade 3 gastroenteropancreatic neuroendocrine neoplasms: morphological differentiation and proliferation identify different prognostic categories. Neuroendocrinol (2017) 104(1):85–93. doi: 10.1159/000445165 26943788

[8] Lithgow K Venkataraman H Hughes S Shah H Kemp-Blake J Vickrage S. Well-differentiated gastroenteropancreatic G3 NET: findings from a large single centre cohort. Sci Rep (2021) 11(1):17947. doi: 10.1038/s41598-021-97247-x 34504148 PMC8429701

[9] Coriat R Walter T Terris B Couvelard A Ruszniewski P. Gastroenteropancreatic well-differentiated grade 3 neuroendocrine tumors: review and position statement. Oncologist (2016) 21(10):1191–9. doi: 10.1634/theoncologist.2015-0476 PMC5061528 27401895

[10] Basturk O Yang Z Tang LH Hruban RH Adsay V McCall CM. The high-grade (WHO G3) pancreatic neuroendocrine tumor category is morphologically and biologically heterogenous and includes both well differentiated and poorly differentiated neoplasms. Am J Surg Pathol (2015) 39(5):683–90. doi: 10.1097/PAS.0000000000000408 PMC4398606 25723112

[11] Vélayoudom-Céphise FL Duvillard P Foucan L Hadoux J Chougnet CN Leboulleux S. Are G3 ENETS neuroendocrine neoplasms heterogeneous? Endocr Relat Cancer (2013) 20(5):649–57. doi: 10.1530/ERC-13-0027 23845449

[12] Popper H. Pathologic diagnosis of lung cancer - recent developments. Curr Opin Oncol (2023) 36(1):57–62. doi: 10.1097/CCO.0000000000001011 37975321

[13] Basturk O Tang L Hruban RH Adsay V Yang Z Krasinskas AM. Poorly differentiated neuroendocrine carcinomas of the pancreas: a clinicopathologic analysis of 44 cases. Am J Surg Pathol (2014) 38(4):437–47. doi: 10.1097/PAS.0000000000000169 PMC3977000 24503751

[14] Scoazec JY CA Monges G Leteurtre E Belleannee G Guyetant S Duvillard P. Well-differentiated grade 3 digestive neuroendocrine tumors: myth or reality? The PRONET Study Group. J Clin Oncol (2012) 30(Supplement). doi: 10.1200/jco.2012.30.15_suppl.4129

[15] Tang LH Untch BR Reidy DL O'Reilly E Dhall D Jih L. Well-differentiated neuroendocrine tumors with a morphologically apparent high-grade component: A pathway distinct from poorly differentiated neuroendocrine carcinomas. Clin Cancer Res (2016) 22(4):1011–7. doi: 10.1158/1078-0432.CCR-15-0548 PMC4988130 26482044

[16] Ziogas IA Tasoudis PT Borbon LC Sherman SK Breheny PJ Chandrasekharan C. Surgical management of G3 gastroenteropancreatic neuroendocrine neoplasms: A systematic review and meta-analysis. Ann Surg Oncol (2023) 30(1):148–60. doi: 10.1245/s10434-022-12643-5 36227392

[17] Holmager P Langer SW Kjaer A Ringholm L Garbyal RS Pommergaard HC. Surgery in patients with gastro-entero-pancreatic neuroendocrine carcinomas, neuroendocrine tumors G3 and high grade mixed neuroendocrine-non-neuroendocrine neoplasms. Curr Treat Options Oncol (2022) 23(6):806–17. doi: 10.1007/s11864-022-00969-x 35362798

[18] Sorbye H Welin S Langer SW Vestermark LW Holt N Osterlund P. Predictive and prognostic factors for treatment and survival in 305 patients with advanced gastrointestinal neuroendocrine carcinoma (WHO G3): the NORDIC NEC study. Ann Oncol (2013) 24(1):152–60. doi: 10.1093/annonc/mds276 22967994

[19] Liu AJ Ueberroth BE McGarrah PW Buckner Petty SA Kendi AT Starr J. Treatment outcomes of well-differentiated high-grade neuroendocrine tumors. Oncologist (2021) 26(5):383–8. doi: 10.1002/onco.13686 PMC8100548 33496040

[20] Apostolidis L Dal Buono A Merola E Jann H Jäger D Wiedenmann B. Multicenter analysis of treatment outcomes for systemic therapy in well differentiated grade 3 neuroendocrine tumors (NET G3). Cancers (Basel) (2021) 13(8). doi: 10.3390/cancers13081936 PMC8073753 33923759

[21] Sonbol MB Halfdanarson TR. Management of well-differentiated high-grade (G3) neuroendocrine tumors. Curr Treat Options Oncol (2019) 20(9):74. doi: 10.1007/s11864-019-0670-1 31428952

[22] McGarrah PW HT Starr JS Kendi AT Graham RP Sonbol MB Halfdanarson TR. Efficacy of somatostatin analog (SSA) monotherapy for well-differentiated grade 3 (G3) gastroenteropancreatic neuroendocrine tumors (NETs). . J Clin Oncol (2020) 38(4). doi: 10.1200/JCO.2020.38.4_suppl.617

[23] Strosberg J El-Haddad G Wolin E Hendifar A Yao J Chasen B. Phase 3 trial of (177)Lu-dotatate for midgut neuroendocrine tumors. N Engl J Med (2017) 376(2):125–35. doi: 10.1056/NEJMoa1607427 PMC5895095 28076709

[24] Thang SP Lung MS Kong G Hofman MS Callahan J Michael M. Peptide receptor radionuclide therapy (PRRT) in European Neuroendocrine Tumour Society (ENETS) grade 3 (G3) neuroendocrine neoplasia (NEN) - a single-institution retrospective analysis. Eur J Nucl Med Mol Imaging (2018) 45(2):262–77. doi: 10.1007/s00259-017-3821-2 28894897

[25] Carlsen EA Fazio N Granberg D Grozinsky-Glasberg S Ahmadzadehfar H Grana CM. Peptide receptor radionuclide therapy in gastroenteropancreatic NEN G3: a multicenter cohort study. Endocr Relat Cancer (2019) 26(2):227–39. doi: 10.1530/ERC-18-0424 30540557

[26] Panzuto F Rinzivillo M Spada F Antonuzzo L Ibrahim T Campana D. Everolimus in pancreatic neuroendocrine carcinomas G3. Pancreas (2017) 46(3):302–5. doi: 10.1097/MPA.0000000000000762 28099254

[27] Pellat A Dreyer C Couffignal C Walter T Lombard-Bohas C Niccoli P. Clinical and biomarker evaluations of sunitinib in patients with grade 3 digestive neuroendocrine neoplasms. Neuroendocrinol (2018) 107(1):24–31. doi: 10.1159/000487237 29518779

[28] Mizuno Y Kudo A Akashi T Akahoshi K Ogura T Ogawa K. Sunitinib shrinks NET-G3 pancreatic neuroendocrine neoplasms. J Cancer Res Clin Oncol (2018) 144(6):1155–63. doi: 10.1007/s00432-018-2636-2 PMC11813331 29602973

[29] Patel SP Othus M Chae YK Giles FJ Hansel DE Singh PP. A phase II basket trial of dual anti-CTLA-4 and anti-PD-1 blockade in rare tumors (DART SWOG 1609) in patients with nonpancreatic neuroendocrine tumors. Clin Cancer Res (2020) 26(10):2290–6. doi: 10.1158/1078-0432.CCR-19-3356 PMC7231627 31969335

[30] Al-Toubah T Halfdanarson T Gile J Morse B Sommerer K Strosberg J. Efficacy of ipilimumab and nivolumab in patients with high-grade neuroendocrine neoplasms. ESMO Open (2022) 7(1):100364. doi: 10.1016/j.esmoop.2021.100364 34973511 PMC8728436

[31] Oronsky B Ma PC Morgensztern D Carter CA. Nothing but NET: A review of neuroendocrine tumors and carcinomas. Neoplasia (2017) 19(12):991–1002. doi: 10.1016/j.neo.2017.09.002 29091800 PMC5678742

[32] Eto K Yoshida N Iwagami S Iwatsuki M Baba H. Surgical treatment for gastrointestinal neuroendocrine tumors. Ann Gastroenterol Surg (2020) 4(6):652–9. doi: 10.1002/ags3.12396 PMC7726685 33319155

[33] Yang M Zeng L Hou S Tian B Jin S Zhang Y. Surgical outcomes, long-term survivals and staging systems of world health organization G3 pancreatic neuroendocrine tumors. J Clin Med (2022) 11(18). doi: 10.3390/jcm11185253 PMC9502090 36142900

[34] Chakedis J Schmidt CR. Surgical treatment of metastatic colorectal cancer. Surg Oncol Clin N Am (2018) 27(2):377–99. doi: 10.1016/j.soc.2017.11.010 29496096

[35] Jamison RL Donohue JH Nagorney DM Rosen CB Harmsen WS Ilstrup DM. Hepatic resection for metastatic colorectal cancer results in cure for some patients. Arch Surg (1997) 132(5):505–10. doi: 10.1001/archsurg.1997.01430290051008 9161393

[36] Wigge S Heißner K Steger V Ladurner R Traub F Sipos B. Impact of surgery in patients with metastatic soft tissue sarcoma: A monocentric retrospective analysis. J Surg Oncol (2018) 118(1):167–76. doi: 10.1002/jso.25115 PMC6668010 29953623

[37] Bristow RE Tomacruz RS Armstrong DK Trimble EL Montz FJ. Survival effect of maximal cytoreductive surgery for advanced ovarian carcinoma during the platinum era: a meta-analysis. J Clin Oncol (2002) 20(5):1248–59. doi: 10.1200/JCO.2002.20.5.1248 11870167

[38] Reuss A du Bois A Harter P Fotopoulou C Sehouli J Aletti G. TRUST: Trial of Radical Upfront Surgical Therapy in advanced ovarian cancer (ENGOT ov33/AGO-OVAR OP7). Int J Gynecol Cancer (2019) 29(8):1327–31. doi: 10.1136/ijgc-2019-000682 31420412

[39] Aparicio T Ducreux M Baudin E Sabourin JC De Baere T Mitry E. Antitumour activity of somatostatin analogues in progressive metastatic neuroendocrine tumours. Eur J Cancer (2001) 37(8):1014–9. doi: 10.1016/S0959-8049(01)00073-9 11334727

[40] Alevroudis E Spei ME Chatziioannou SN Tsoli M Wallin G Kaltsas G. Clinical utility of (18)F-FDG PET in neuroendocrine tumors prior to peptide receptor radionuclide therapy: A systematic review and meta-analysis. Cancers (Basel) (2021) 13(8). doi: 10.3390/cancers13081813 PMC8069875 33920195

[41] Uccella S La Rosa S Metovic J Marchiori D Scoazec JY Volante M. Genomics of high-grade neuroendocrine neoplasms: well-differentiated neuroendocrine tumor with high-grade features (G3 NET) and neuroendocrine carcinomas (NEC) of various anatomic sites. Endocr Pathol (2021) 32(1):192–210. doi: 10.1007/s12022-020-09660-z 33433884

[42] Venizelos A Elvebakken H Perren A Nikolaienko O Deng W Lothe IMB. The molecular characteristics of high-grade gastroenteropancreatic neuroendocrine neoplasms. Endocr Relat Cancer (2021) 29(1):1–14. 34647903 10.1530/ERC-21-0152 PMC8630776

