# Supplementary figures and images for: Treatment modalities favoring outcome in well-differentiated neuroendocrine tumors G3
Source: Front Endocrinol (Lausanne). 2024 Jan 8;14:1285529. doi: 10.3389/fendo.2023.1285529 (PMC10800837; doi:10.3389/fendo.2023.1285529)

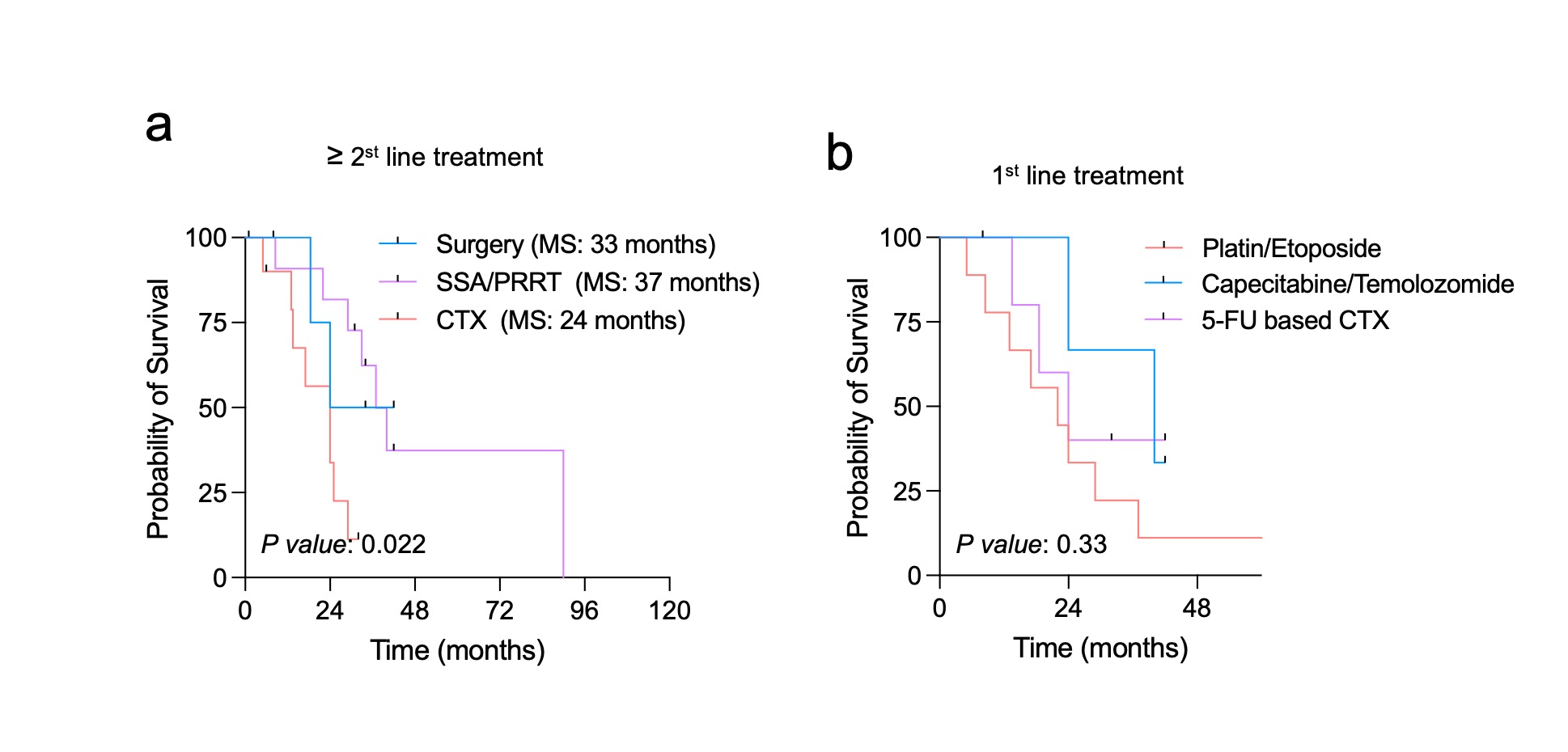

Supplement: Supplementary Figure 1 — Treatment outcomes in NET G3. (A) Kaplan–Meier curves estimates of OS (in months) in patients receiving surgery, SSA/PRRT or CTX in ≥ second line treatment. (B) Kaplan–Meier curves estimates of OS (in months) in NET G3 patients receiving CTX. [file Image_1.jpeg]

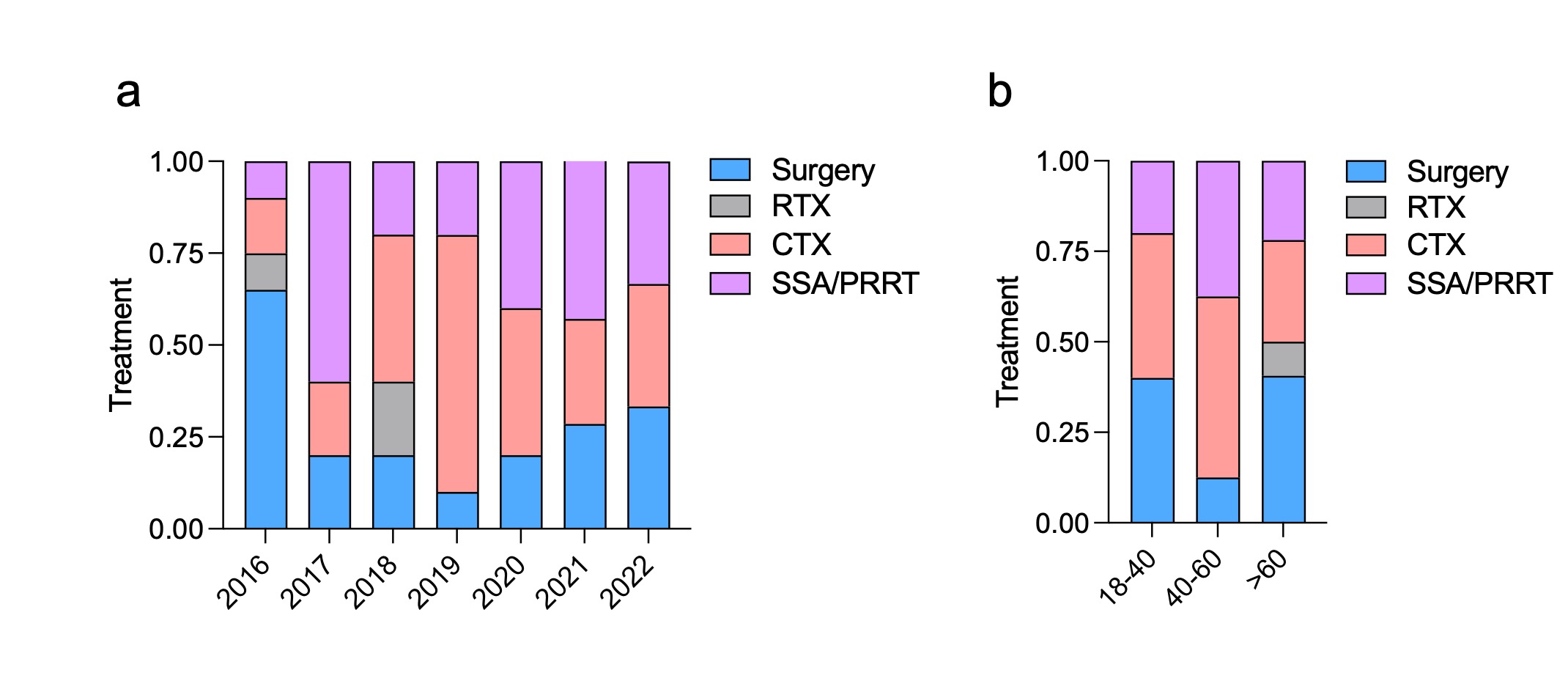

Supplement: Supplementary Figure 2 — Distribution of treatment modalities over time and patient age. (A) Distribution of treatment modalities over time. (B) Correlation of treatment modalities and patient age. [file Image_2.jpeg]

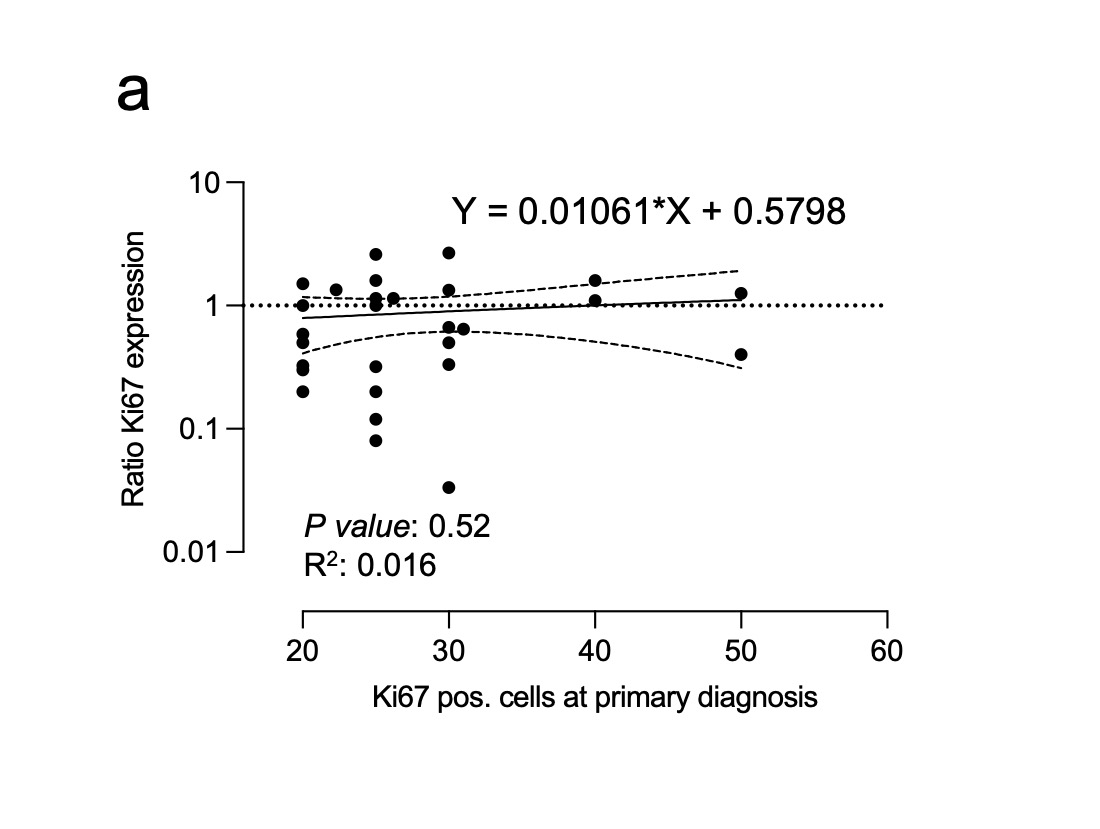

Supplement: Supplementary Figure 3 — Changes in Ki67 levels in NET G3. (A) Correlation of Ki67 levels at primary diagnosis and Ki67 ratios. [file Image_3.jpeg]
