# Supplementary material for: Treatment modalities favoring outcome in well-differentiated neuroendocrine tumors G3
Source: Front Endocrinol (Lausanne). 2024 Jan 8;14:1285529. doi: 10.3389/fendo.2023.1285529 (PMC10800837; doi:10.3389/fendo.2023.1285529)
Supplement: Supplementary file 4 [file Table_1.docx]

**Supplementary Table 1: Treatment schemes of the NET G3 cohort in further therapy lines.**

5-FU = 5-fluorouracil, ACO = adriamycin, vincristine, cyclophosphamide, ADOC = doxorubicin, cisplatin, vincristine, cyclophosphamide, ATR = Ataxia telangiectasia and Rad3 related, CAPTEM = capecitabine/temozolomide, FLOT = 5-fluorouracil, leucovorin, oxaliplatin, docetaxel, FOLFIRI = irinotecan, 5-fluorouracil, leucovorin, FOLFOX = oxaliplatin, 5-fluorouracil, leucovorin, G3 = grading 3, HDAC = histone deacetylase, n = number, NET = neuroendocrine tumor, PRRT = peptide receptor radionucleotide therapy, RT = radiotherapy, SIRT = selective internal radiotherapy, SSA = somatostatin analogue, SSTR = somatostatin receptor, TACE = transarterial chemoembolization.

| **Treatment schemes** | **Patients** |
| --- | --- |
| **Further therapy line regimen, n** | maximum of 13 therapy lines in 2 patients |
| Surgery of the primary tumor site | 1 |
| Surgery of metastases | 6 |
| SSA | 1 |
| PRRT | 10, including 6 in combination with radiosensitizing (CAPTEM) |
| SIRT | 7 |
| TACE | 2 |
| RT of metastases | 16 |
| Systemic therapies (other than SSTR-directed) | 56 |
| Platinum/etoposide | 10 |
| CAPTEM | 9 |
| Capecitabine/temozolomid/vorinostat (HDAC inhibitor) | 1 |
| FOLFOX | 1 |
| FOLFOX/bevacizumab | 2 |
| FOLFIRI | 4 |
| 5-FU | 1 |
| 5-FU/bevacizumab | 2 |
| FLOT | 1 |
| Topotecan | 3 |
| Topotecan/olaparib | 1 |
| Streptozotocin/5-FU | 2 |
| Carboplatin/gemcitabine | 1 |
| Carboplatin/irinotecan | 1 |
| Carboplatin/ATR inhibitor | 1 |
| Docetaxel/Ramucirumab | 1 |
| Cyclophosphamid | 1 |
| ACO | 1 |
| ADOC | 1 |
| Everolimus | 3 |
| Everolimus/lenvatinib | 1 |
| Sunitinib | 2 |
| Sorafenib | 1 |
| Afatinib | 1 |
| Ponatinib | 1 |
| Olaparib | 1 |
| Olaparib/ATR inhibitor | 1 |
| Nivolumab/ipilimumab | 1 |
| Nivolumab | 1 |
| Pembrolizumab/lenvatinib | 1 |
